# Next-Generation Hydrogels for Biliary Organoid Engineering

**DOI:** 10.3390/ph18121781

**Published:** 2025-11-23

**Authors:** Andrea Marfoglia, Giovanni Sorrentino

**Affiliations:** 1 Department of Medical, Surgical and Health Sciences, University of Trieste, Strada di Fiume 477, 34139 Trieste, Italy; 2 International Centre for Genetic Engineering and Biotechnology (ICGEB), Area Science Park-Padriciano, 34149 Trieste, Italy

**Keywords:** biliary organoids, bile ducts, cholangiopathies, hydrogels, biomechanics, disease models, tissue engineering, regenerative medicine, biomaterials, liver

## Abstract

The biliary tree is a fundamental structural and functional component of the liver, lined with cholangiocytes which control bile flow and regulate bile homeostasis. In addition to their physiological roles, cholangiocytes are involved in pathological processes known as cholangiopathies. These biliary disorders significantly impair liver function, and their effects are often irreversible, making liver transplantation the only curative option. This substantial clinical burden highlights the need for innovative bioengineered strategies to study disease mechanisms and to restore or replace biliary tissue. In this framework, biliary organoids offer a robust platform to model liver diseases in vitro with physiological accuracy. Compared with traditional 2D or explant-based systems, organoids provide higher physiological relevance, patient specificity, and scalability, although challenges remain in standardization and clinical translation. Organoids are traditionally cultured within basement membrane extract (BME) matrices, which are commercially available under various names. While BME-based matrices support organoid growth and function, their undefined composition, variability, and animal origin limit reproducibility and clinical translation. These drawbacks have driven the development of alternative matrices based on engineered hydrogels. Hydrogels, whether of natural or synthetic origin, provide chemically defined and tunable environments that allow independent modulation of their biochemical and biophysical properties. Acting at the interface between materials science and biology, they enable the creation of microenvironments with precisely controlled cues. In this review, we summarize advances in biliary organoid bioengineering and discuss how hydrogel-based systems are shaping next-generation platforms for organoid growth, differentiation, and disease modeling toward more translationally relevant biliary models.

## 1. Introduction: Developmental and Pathological Landscape of the Biliary Tree

The biliary system is a branched network of ducts that channels bile from the liver into the intestine to aid in fat digestion and remove waste from the liver. It comprises the intrahepatic bile ducts (IHBDs) in the liver parenchyma and the extrahepatic bile ducts (EHBDs), such as the common hepatic duct, the cystic duct, and the gallbladder [1]. These ducts are lined with cholangiocytes, epithelial cells with specific absorptive and transport functions essential for bile modification and homeostasis [2]. Embryologically, the biliary tree originates from the ventral foregut endoderm, where early progenitors diverge into intrahepatic and extrahepatic lineages, establishing the anatomical and functional compartmentalization of the biliary system [3,4]. In adulthood, the biliary epithelium shows regional heterogeneity with cholangiocyte subtypes displaying different molecular profiles, proliferative capacities, and injury responses, a feature increasingly recognized in models of liver injury and regeneration [5]. This mosaic of cholangiocyte states is crucial for bile modification and repair processes. Within the canal of Hering, the structure connecting bile canaliculi to bile ducts, liver progenitor cells (LPCs) are found. LPCs are adult bipotent stem cells characterized by remarkable plasticity. They express cholangiocyte markers (EpCAM, KRT19, KRT7) as well as hepatocyte markers (albumin, KRT18, KRT8) and then differentiate into both cell types in response to injury [6]. Interestingly, the origin of LPCs was traced back to both cholangiocytes and hepatocytes, which actively participate in the regeneration of the liver parenchyma [7,8,9].

Cholangiopathies include a range of diseases that selectively target cholangiocytes and the biliary system, often interfering with the production, secretion, and transport of bile. In this context, bile components accumulate within the liver parenchyma, leading to bile spillover, hepatocellular toxicity, and cholestasis [10,11]. In cholangiopathies, biliary cells are not passive targets of liver damage but are actively involved in the development and progression of these diseases [12]. In response to extensive parenchymal damage or when exposed to genetic mutations, infections, immune-mediated injury, or vascular insults, cholangiocytes and LPCs undergo profound phenotypic changes that lead to an activated, or “reactive,” state. In this condition, they proliferate, infiltrate into the parenchyma, and secrete proinflammatory cytokines and chemokines that recruit immune cells and modulate neighboring stromal and immune populations [13]. This coordinated epithelial and stromal response is known as the ductular reaction (DR), a hallmark of chronic biliary diseases. The DR represents an essential regenerative mechanism that compensates for biliary cell loss or, in the context of parenchymal injury, insufficient hepatocyte replication; however, when persistent in chronic diseases, it becomes maladaptive, sustaining inflammation, matrix deposition, and tissue remodeling [14]. The delicate balance between regeneration and pathology in DR highlights the central role of cholangiocytes in both homeostasis and the progression of liver diseases. Several paradigmatic cholangiopathies, such as primary sclerosing cholangitis (PSC), biliary atresia (BA) and Alagille syndrome (ALGS) illustrate the complexity of these processes [15,16,17]. Collectively, these disorders illustrate how chronic cholangiocyte injury and the resulting ductular reaction create a pro-inflammatory, fibrogenic microenvironment marked by altered extracellular matrix composition and aberrant biochemical cues that perpetuate stromal and parenchymal activation [18,19,20,21].

In PSC, a fibroinflammatory disease of immune-mediated origin, chronic inflammation and bile duct strictures progressively damage the intrahepatic and extrahepatic biliary tree. Currently, there are no curative therapies other than liver transplantation [22]. BA is an obliterative disease of infancy that affects the extrahepatic ducts and leads to their progressive loss, resulting in inflammation and fibrosis at an early age [23]. Eventually, 60–75% of patients require liver transplant due to the progressive nature of the disease [24]. ALGS is a disease affecting multiple organs caused by impaired Notch signaling. In the liver, it results in a reduced number of bile ducts and progressive cholestasis [25,26]. Although often underrepresented in mechanistic studies, ALGS provides a valuable model for dissecting the developmental cues that shape biliary tree architecture. The great majority of ALGS cases are associated with mutations or deletions in the *JAG1* gene, which encodes the Notch ligand Jagged, while only about 1% of cases are related to mutations in the *NOTCH2* gene [25,27]. Cystic fibrosis (CF) is a multi-organ disease caused by mutations in the *CFTR* gene, leading to progressive organ damage. In cholangiocytes, this results in impaired alkalinization of bile, causing ductal obstruction and periportal fibrosis [28]. Polycystic liver disease (PLD), often associated with ductal plate malformations and mutations in ciliary proteins, results in abnormal proliferation of cholangiocytes, which promote cystogenesis and parenchymal distortion [29].

Within this pathological continuum, cholangiocarcinoma (CCA) emerges as the malignant culmination of these chronic regenerative and fibrogenic processes [30]. CCA can emerge from any region of the biliary tree and is classified as intrahepatic or extrahepatic, depending on the anatomical site of origin [31]. Importantly, ductular reactive cells have been described as major cells of origin for CCA, underscoring the central role of chronic biliary activation in tumorigenesis [32,33]. In line with this assumption, PSC—characterized by sustained DR—is a well-established risk factor for CCA onset [34]. The prognosis for CCA is usually poor because the early stages of the disease are asymptomatic, which delays diagnosis. Additionally, CCAs are desmoplastic due to increased collagen deposition by cancer-associated fibroblasts, resulting in a stiff mass that provides critical biomechanical cues to promote tumor progression and immune surveillance elusion [35,36,37].

## 2. Biliary Organoids: From Disease Modeling to Regenerative Medicine

**Figure 1:**
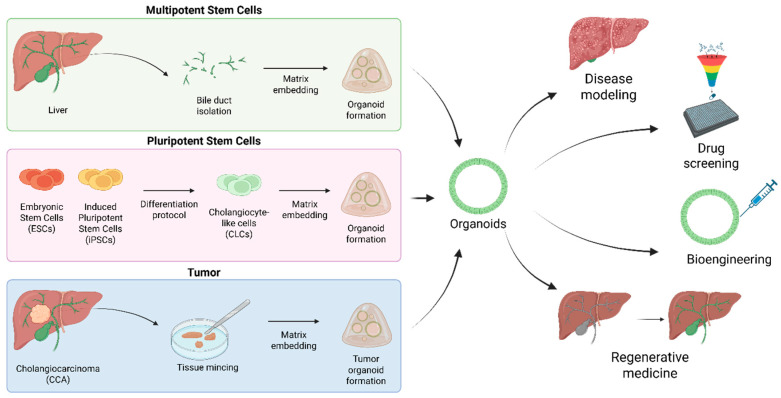
Biliary organoids: from disease modeling to regenerative medicine. Biliary organoids can be established from multiple sources, including primary bile ducts isolated from liver tissue, pluripotent stem cells (ESCs and iPSCs) differentiated into cholangiocyte-like cells (CLCs), and tumor biopsies or resections from cholangiocarcinoma (CCA) patients. In each case, cells are embedded within a suitable matrix to promote self-organization and cystic organoid formation. These biliary organoids recapitulate the structural and functional features of native bile ducts and can be used as in vitro models for disease modeling, drug screening, and regenerative medicine. Applications include the reconstruction of damaged bile ducts, functional assays, and patient-derived organoid models for precision oncology. Created in BioRender. Sorrentino, G. (2025) https://BioRender.com/9ym0xl3 (accessed on 14 November 2025).

Biliary organoids are self-organized three-dimensional (3D) structures that recapitulate in vitro the architecture and functions of bile ducts [38]. Biliary organoids can be derived from both embryonic stem cells and induced pluripotent stem cells (ESCs and iPSCs, respectively) [39,40,41,42,43] or directly from adult tissues and bile [44,45,46] (Figure 1). In the latter, biliary organoids are typically established by isolating bile ducts fragments or EpCAM+ adult progenitor/stem cells from the intrahepatic bile ducts by mechanical and/or enzymatic dissociation [45,46]. These cells are then embedded in basement membrane extracts (BME), commercially available under different names such as Matrigel or Cultrex, where they self-organize into 3D organoids, under Wnt/β-catenin stimulating culture conditions [47]. These biliary organoids were demonstrated to expand long term in culture, maintaining genetic stability over time in contrast with classical 2D cell cultures which often acquire de novo TP53 mutations. Owing to the bipotent nature of these cells, these organoids can be further differentiated into both cholangiocyte-like and hepatocyte-like cells by changing culture conditions [45,48,49].

Cholangiocyte-like cells (CLCs) organoids derived from ESCs/iPSCs are instead generated by sequential differentiation protocols that mimic embryonic development. In vivo, cholangiocytes and hepatocytes originate from hepatoblasts [50]. Accordingly, iPSCs are first differentiated into foregut progenitor cells and subsequently into bipotent hepatoblasts. To direct hepatoblasts toward cholangiocyte fate, culture conditions that promote cholangiocyte specification are required. Sampaziotis et al. developed a stepwise differentiation protocol in which retinoic acid and Activin A suppress hepatocyte markers, while stimulation with fibroblast growth factor 10 (FGF10) upregulates biliary specification genes such as *SOX9*, *HNF1B* and *CK19* [41]. Under these conditions, hepatoblasts acquire a cholangiocyte-progenitor phenotype. Finally, removal of the inductive factors and addition of epidermal growth factor (EGF), combined with embedding the cells in BME support 3D morphogenesis and maturation into cholangiocyte organoids. Organoids obtained from these methods reproduce key functions of bile ducts, including bile acid transport, and maintain cholangiocyte markers, namely KRT-7 and 19, and EpCAM expression [39,41,45]. In all models, BME serves as a permissive substrate for both expansion and 3D self-organization into duct-like cystic structures.

Biliary organoids provide a robust platform to model disease-relevant phenotypes and study cholangiocyte-intrinsic mechanisms, as they retain in vitro many of the features observed in primary disease [41]. To better understand disease-relevant features, numerous studies have focused on generating organoids directly from patients. Patient-derived organoids from PSC patients, obtained either from their bile via ERCP, reproduce senescence-associated proinflammatory signatures [51]. Similarly, organoids derived from BA patients recapitulate hallmarks such as aberrant morphology and loss of apicobasal polarity, and have uncovered the previously unrecognized accumulation of beta-amyloid aggregates, demonstrating their utility for identifying novel pathological features [52]. Patient-derived and iPSC-derived organoids have been employed to model CF and PLD, providing a platform to test pharmacological interventions such as elexacaftor/ivacaftor/tezacaftor (ELX/IVA/TEZ) or Octreotide [41,53]. Drug-induced disease modeling has also been applied to normal biliary organoids; for example, exposure to biliatresone induces oxidative and ER stress, recapitulating key features of BA pathology [54,55]. Finally, organoids from murine models of ALGS reveal structural and regional differences in proliferation, Notch signaling, and hepatocyte marker expression, while exposure to bile acids can partially re-establish region-specific identities [56,57]. Human biliary organoids have been used to model the emergence of biphenotypic cells co-expressing hepatocyte and cholangiocyte markers, mirroring those observed in chronic human liver disease [58]. These findings highlight that biliary organoids retain a plastic and environmentally responsive phenotype, capable of reacquiring regional identity under appropriate niche cues and capturing epithelial plasticity as well as disease-associated shifts in cell identity.

Organoids derived from cholangiocarcinoma (CCA) biopsies provide patient-specific models to investigate tumor heterogeneity and drug sensitivity (Figure 1) [59,60]. Li et al. demonstrated their translational value by screening 129 compounds across multiple patient-derived CCA organoid lines, revealing marked intra- and inter-patient variability in drug responses [61]. Beyond patient-derived systems, genetically engineered organoids have been used to model key oncogenic drivers. IDH1^R132C^-mutant organoids displayed growth advantages under growth factor–deprived conditions, while FGFR2 fusion–expressing Tp53^−^/^−^ organoids generated desmoplastic CCA-like tumors in vivo, capturing histopathological features of the disease [62,63]. Recently, culture optimization enabled the generation of branched cholangiocyte and CCA organoids that more closely resemble primary tissue and tumor in morphology, transcriptome, and drug resistance profiles [64]. Together, these models offer versatile platforms for dissecting CCA pathogenesis, mapping tumor heterogeneity, and predicting therapeutic responses with clinical relevance.

The liver is the only organ capable of fully regenerating itself to maintain the liver-to-body weight ratio required for homeostasis. However, this regenerative capacity can be severely impaired by a plethora of pathological conditions, making organ transplantation the only therapeutic solution. In this context, organoids have attracted attention due to their regenerative potential, as they preserve important functions of their tissue of origin. In mice, Sampaziotis et al. have shown that organoids derived from the gallbladder can repair irreversibly damaged bile ducts [65]. Moreover, the authors were also able to replicate engraftment on a human liver ex vivo. In another study, Huch and colleagues were able to culture human biliary organoids and differentiate them into hepatocytes to transplant them into a hepatotoxic mouse model [46]. After transplantation, the organoids engrafted into the mouse liver and exhibited hepatocyte markers.

Altogether, these studies elevate biliary organoids as critical tools for disease modeling and tissue engineering.

## 3. Hydrogels: The Next Frontier for Biliary Organoid Engineering

Advances in bioengineering have increasingly highlighted the potential of tailored hydrogel systems to recreate physiologically relevant microenvironments for biliary organoid culture [66,67]. By enabling the modulation of biochemical and mechanical cues, these materials can support more faithful modeling of biliary tissue organization, regeneration, and disease mechanisms [68,69]. However, despite the growing availability of engineered matrices, most current organoid studies still rely on BME, which presents several intrinsic limitations. Understanding these limitations is essential to appreciate the need for next-generation hydrogel platforms.

BME is currently the gold standard for organoid cultures. It is derived from mouse Englebreth-Holm-Swarm (EHS) sarcoma subcutaneous tumors and is a laminin-rich hydrogel that provides a favorable microenvironment for cell proliferation and self-organization into 3D structures [70]. Despite their utility and widespread use, BME-based hydrogels have significant limitations. Their composition is non-tissue-specific, complex, poorly defined, and often subject to lot-to-lot variation, which complicates reproducibility. Furthermore, because BME is animal-derived, concerns about its antigenicity strongly limit the potential use of organoids grown in BME for clinical applications [71]. In experimental settings, this complicates the distinction between biological effects arising from controlled variables and those inadvertently introduced by components of the BME. Moreover, the physical and mechanical properties of BME hydrogels cannot be easily uncoupled from their strong biochemical signals, owing to the presence of more than 1800 proteins [72]. Additionally, heterogeneous mechanical cues were identified within the same BME hydrogel, with local stiffness varying severalfold across different regions [73,74]. Since mechanical cues are now established as important modulators of cell behavior, the inability to precisely control these parameters hinders biomechanical studies of organoids grown in BME [75,76]. Although it could be argued that varying BME concentration allows for partial tuning of the stiffness of BME, biochemical properties, such as integrin ligands density, are inevitably affected. Moreover, these hydrogels struggle to reach physiologically relevant stiffnesses. This is particularly important considering that it is well known that many pathological conditions induce abnormal ECM architecture and composition which, in turn, dysregulates mechanical properties and consequently, mechanotransduction [21,77,78,79,80,81,82].

As an alternative to BME, an ideal platform for biliary organoid culture would be represented by hydrogels that fulfill several key criteria, including a clearly defined biochemical composition, independently tunable stiffness and viscoelasticity, robust support for organoid formation and differentiation, and compatibility with clinical translation [83]. Hydrogels are polymer networks capable of retaining water or other interstitial fluids [84]. Various polymers can be used to produce hydrogels, allowing them to be tailored to research needs. Most hydrogel systems allow some tuning of their mechanical properties, porosity, bioactivity, and other factors through different strategies. Based on their origin, hydrogels can be classified as either natural or synthetic, and natural polymers can be modified to produce semi-synthetic variants with improved tunability, bioactivity, and performance [85]. Additionally, composite hydrogels, namely hydrogels prepared by mixing natural and synthetic polymers together, are often used to complement each other’s limitations. Thus, hydrogels, with their well-defined biochemical composition and tunable mechanical properties, represent promising platforms bridging experimental modeling and clinical translation [86,87].

### 3.1. Mechanical Properties of Hydrogels

**Figure 2:**
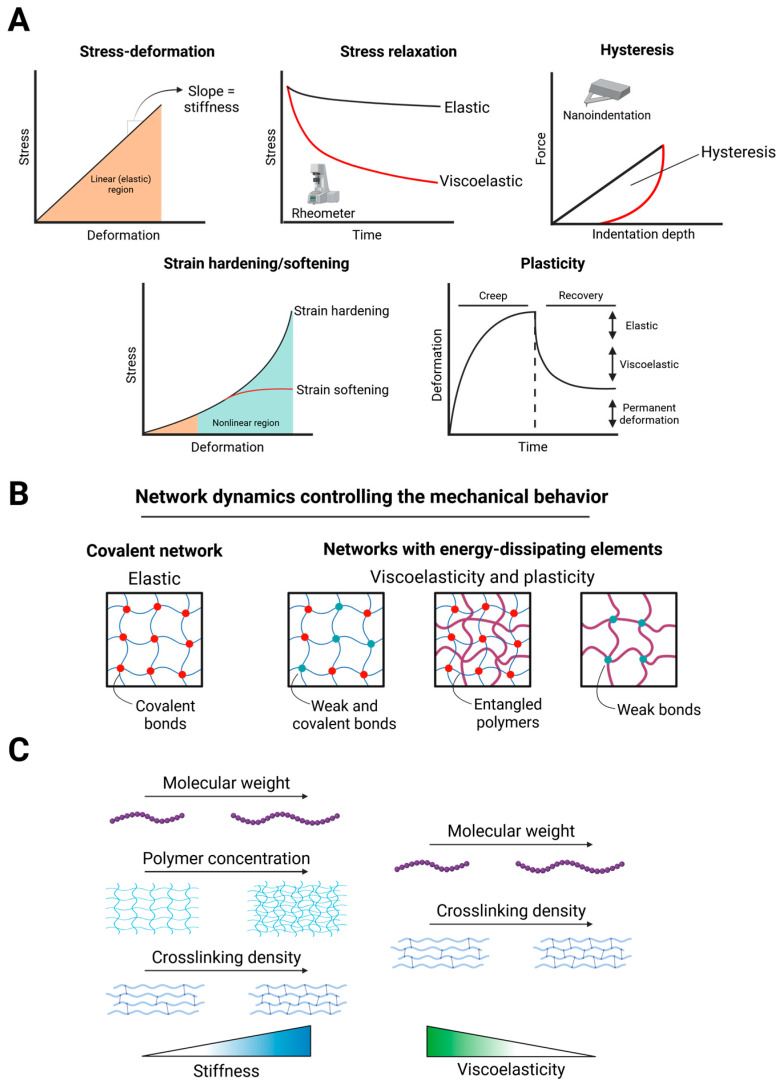
Schematic representation of the key mechanical properties and molecular architectures governing hydrogel behavior. (**A**) Linear elastic materials exhibit a proportional stress–strain relationship within the elastic region, whereas biological and polymeric networks may display non-linear responses such as strain hardening (stiffening upon deformation) or strain softening. Viscoelastic materials dissipate energy over time, as shown by stress relaxation or hysteresis under cyclic loading. Plastic materials undergo irreversible deformation beyond the yield point. (**B**) Crosslinking chemistry determines the mechanics of hydrogels: covalent crosslinks generate purely elastic networks, whereas reversible (i.e., ionic) crosslinks confer viscoelastic or viscoplastic behavior. (**C**) The degree of crosslinking, polymer molecular weight, and network density can be tuned to control stiffness and relaxation times. Together, these principles define how hydrogel mechanics emerge from the underlying polymer structure and crosslinking dynamics, ultimately influencing cell mechanotransduction and organoid morphogenesis. Created in BioRender. Sorrentino, G. (2025) https://BioRender.com/9ym0xl3 (accessed on 14 November 2025).

Mechanical properties of tissues and hydrogels in the laboratory setting are most often determined through either rheology or compression techniques, such as nanoindentation (Figure 2A). The stiffness, or linear elasticity, of materials is commonly determined and is historically the most studied biomechanical variable. It is described by the stress–strain response of a material and referred to as Young’s (elastic) modulus if obtained through compression, or shear modulus if obtained via rheometry. Viscoelastic parameters are described by stress relaxation behavior (rheology) and hysteresis area (nanoindentation). When deformed, viscoelastic materials rearrange to dissipate stress over time, opposed to purely elastic materials which store stress (Figure 2A). While stiffness has long been described to strongly influence cellular behavior, only more recently viscoelasticity is being explored as a critical parameter that regulates spatiotemporal organization of cells and tissues [75,88]. Both stiffness and viscoelasticity are obtained from the linear regime of a material, yet evidence suggests that cells can also access the nonlinear regime. Indeed, plasticity and nonlinear elasticity, also known as strain hardening, are features present in tissues and biological molecules [89,90,91,92,93]. Both natural and synthetic fibrous polymers display a strain hardening behavior, namely they increase in stiffness as the deformation increases, and is opposed to a strain softening behavior, in which the material stiffness decreases as the deformation increases [94,95]. However, the biological impact of strain hardening on cell behavior remains largely unexplored, largely due to the difficulty of modulating it. A similar knowledge gap exists for plasticity, even though more studies, both in 2D and 3D cell culture systems, are present [96,97]. A recent comprehensive review provides an in-depth discussion of the biological relevance of non-linear mechanics in cell behavior, and readers are referred to it for further details [98].

Generally, the hydrogel crosslinking mechanism determines the resulting mechanical behavior [99] (Figure 2B). Stiffness can be modulated by varying the polymer concentration, crosslinking density, or polymer molecular weight (Figure 2C). Ideal covalent networks (i.e., polymers crosslinked through covalent bonds) are elastic; viscoelasticity can instead be introduced by adding weak bonds (for example, ionic bonds), by polymer entanglement events, or by changing the total number of crosslinks [75] (Figure 2C). These features allow the network to dissipate energy and exhibit stress relaxation. Instead, networks crosslinked only through weak bonds can undergo permanent deformation under stress, exhibiting viscoplastic features [75]. Plasticity and viscoelasticity are not mutually exclusive, and hydrogels may exhibit both. Moreover, both plasticity and viscoelasticity depend not only on crosslinking dynamics but also on polymer molecular weight. An example of hydrogels that illustrate these concepts are alginate hydrogels of varying molecular weights prepared with different crosslinker concentrations [88,100,101]. In these hydrogels, the fast or slow relaxing nature depends on the polymer molecular weight, while stiffness is varied by increasing or decreasing the crosslinker concentration, allowing independent control of both mechanical parameters. Similarly, the plasticity of alginate hydrogels is controlled by the polymer molecular weight: high molecular weight alginate networks are less plastic than low molecular weight alginate networks [96]. An example of viscoelasticity influenced by crosslinking strategy comes from collagen hydrogels: covalent norbornene-mediated crosslinks in collagen hydrogels do not affect their stiffness but increase their relaxation times, making them more elastic compared to plain collagen hydrogels [102].

Owing to their ability to precisely tune these parameters, hydrogels provide an excellent platform to recreate liver-mimicking mechanical niches [66]. The stiffness of healthy human liver has been reported to range from approximately 0.4–2 kPa by rheology and around 2 kPa by atomic force microscopy (AFM), whereas cirrhotic liver stiffness can increase by several times [79,103]. Furthermore, recent evidence indicates that liver viscoelastic properties are altered under pathological conditions, highlighting the need for fully tunable hydrogel systems to systematically modulate material mechanics [79]. Since BME offers limited tunability, hydrogels represent a more versatile platform to engineer biologically inspired matrices, capable of recapitulating both physiologically and pathologically relevant mechanical features and composition. However, in the context of biliary organoids, stiffness remains the most studied physical property, warranting more research on other underexplored mechanical parameters.

### 3.2. Natural and Semi-Synthetic Polymers for Hydrogel Fabrication

Natural polymers, also known as biopolymers, are derived from both animal and plant sources and display different properties depending on their biological sources. Both proteins (polypeptides) and polysaccharides can be used to fabricate hydrogels. These polymers can undergo chemical modifications to produce semi-synthetic hydrogels, which are made from polymers that have been altered to address deficiencies in the native polymer. Semi-synthetic hydrogels are hydrogels whose network is composed of a natural biopolymer that has been partially engineered to improve certain properties. For example, gelatin methacryloyl (GelMA) has been developed to significantly increase the mechanical stability of gelatin at physiological temperatures [104]. Another example is alginate, a polysaccharide derived from brown algae, which can be functionalized with integrin binding peptides to compensate for the lack of cell adhesion sites [105]. Compared to synthetic hydrogels, natural matrices typically offer superior biocompatibility and bioactivity that can be used to mimic biologically relevant matrices in vitro. However, they often form weak hydrogels and may require modification to achieve biologically relevant mechanical properties [106].

#### 3.2.1. Decellularized Liver Extracellular Matrix

Decellularized extracellular matrices (dECM) have been proposed as a clinically suitable alternative to EHS-based hydrogels [107]. As the name suggests, it is obtained from the complete removal of all cellular components, DNA and non-ECM proteins from the tissue as they could induce an immunogenic response. The resulting scaffold can then be either repopulated with cells of interest or further digested, usually by enzymatic digestion, to obtain a polymerizable matrix. Liver dECM scaffolds hold great promise for tissue engineering, as they could help address the shortage of donor organs [108]. Tomofuji et al. were able to decellularize rat livers and repopulate the resulting scaffold with both biliary organoids and primary hepatocytes [109]. Biliary organoids, obtained both from mouse and human livers, were successfully engrafted in the intrahepatic bile ducts of the rat decellularized liver. Krüger and colleagues successfully obtained porcine biliary organoids and differentiated them into hepatocyte-like organoids [110]. The resulting organoids were then capable of colonizing decellularized liver scaffold disks and showed stable expression of hepatocyte markers. Engraftment efficiency was high, although only small scaffold disks were repopulated. Together, these studies show that repopulating an entire liver dECM scaffold is technically possible using only primary biliary organoids as a cell source.

As introduced earlier, decellularized liver matrices can be further digested, usually with pepsin, to obtain dECM hydrogels. In contrast to BME, these hydrogels are tissue-specific, enabling the generation of matrices enriched in ECM components characteristic of the liver. The liver dECM hydrogels were tested by Willemse and colleagues as a platform to culture human biliary organoids and drive their differentiation into hepatocytes [111]. The dECM were obtained from both human and porcine livers and displayed an increased stiffness (~250 and ~650 Pa, respectively) compared to BME (~100 Pa). These hydrogels supported both the culturing as well as the initiation of biliary organoids. However, they displayed some differences compared to BME, as the authors reported the formation of both “branches” and tubular structures, other than cysts. Notably, a previous study had already described the formation of tubular structures from cholangiocytes cultured in the dECM [112]. Biliary organoids were subsequently differentiated into hepatocyte-like cells, achieving maturation levels comparable to those obtained in BME-based cultures. These hydrogels effectively recapitulate the native liver microenvironment, as they preserve the core structural and biochemical components of the extracellular matrix, collectively known as the matrisome [113].

Since CCA presents profound ECM components dysregulation, dECM obtained from these tumors could aid lead to a better understanding of the relevance of the microenvironment in cancer initiation, progression and aggressive behavior [114,115,116]. In this context, van Tienderen and colleagues cultured CCA patient-derived organoids in either healthy liver-derived dECM (dECM-HL) or tumor-derived dECM (dECM-CCA) [117]. Interestingly, the authors found pronounced morphology changes between CCA organoids cultured in either of the dECM or BME. Additionally, the CCA organoids were found to secrete distinct ECM components based on the matrix: while in BME the organoids secreted mostly glycoproteins, such as laminins, organoids cultured in the healthy liver matrix largely increased collagen production compared to the CCA-derived matrix or BME.

Hydrogels based on dECM present however some shortcomings: their formation mostly relies on collagens fibrillogenesis, allowing for limited control over the matrix biophysical properties. For this reason, various authors have described strategies to increase and tune the mechanical properties of dECM hydrogels. For example, in a study by Milton et al., the authors chemically modified liver dECM to fine tune the stiffness of the hydrogels between 1 and 7 kPa, mimicking the healthy and fibrotic liver stiffnesses [118]. Another strategy was proposed by Xue and colleagues to fabricate engineered liver dECM with tunable stiffness and viscoelasticity. By progressively increasing the amount of oxidized dextran used to crosslink the matrix, they were able to modulate both parameters in a controlled manner, enabling the decoupling of cellular responses to mechanical cues within a tissue-relevant ECM environment [119]. Overall, liver dECM provide tissue-specific hydrogels that support biliary organoid growth and differentiation while offering translational potential.

#### 3.2.2. Gelatin and Derivatives

Gelatin is obtained from the hydrolysis of collagen and possesses both RGD sequences and matrix metalloproteinases (MMP)-sensitive sites, so that it enables both cell adhesion and matrix remodeling by cells. Gelatin hydrogels are thermoresponsive, i.e., they turn from gel to liquid when heated to physiological temperatures, which makes their use for cell culture challenging. Various chemical modifications of gelatin yield crosslinkable hydrogels that prevent loss of structure at higher temperatures. One of the most widely used semi-synthetic gelatins is gelatin methacryloyl (GelMA), which can be photocured after the addition of a photoinitiator [104]. In the context of biliary organoids, gelatin derivatives were mainly used to direct biliary organoids to hepatogenic differentiation. In a comprehensive study by Carpentier et al., GelMA, thiolated gelatin (GelSH) and polyisocianopeptide-laminin/entactin complex (PIC-LEC) (PIC-based hydrogels are discussed in a separate section below) were used to culture primary human biliary organoids and differentiate them into hepatocyte-like cells [120]. Additionally, GelSH was further functionalized with either chondroitin sulphate (CS) or dextran (Dex), yielding DexNB-GelSH and CSNB-GelSH, which are photocurable similarly to GelMA [121]. CSNB-GelSH hydrogels with varying degrees of CS functionalization were fabricated to create a liver mimicking matrix, as CS is a major component of the liver ECM. On the other hand, DexNB-GelSH were already reported by the same authors to be optimal in replicating the liver’s mechanical properties [122]. These hydrogels displayed marked differences in mechanical properties which influenced organoid formation and proliferation. The softer hydrogels, namely PIC-LEC (71 Pa) and DexNB-GelSH (6.2 kPa), had reported stiffnesses comparable to that of BME and healthy liver respectively, and supported the expansion of organoids similarly to BME. On the other hand, the stiffer GelMA (9.6 kPa) and CSNB-GelSH (32.5 kPa) hydrogels didn’t support the proliferation of the biliary organoids to the same extent. However, all the gelatin-based hydrogel outperformed both BME and PIC-LEC in directing the organoids towards hepatogenic differentiation. In particular, the stiffer hydrogels in the study improved the differentiation process. Different results were reported by Bouwmeester et al. on how GelMA was not superior, but equal to BME in directing organoids towards a hepatic maturation [123]. In another study, Bernal et al. used GelMA to volumetrically bioprint primary biliary organoids [124]. These organoids were successfully differentiated into hepatocyte-like cells and volumetrically bioprinted to generate liver-like biofactories. By bioprinting specific complex patterns, the organoid-laden constructs performed ammonia detoxification and released liver-specific enzymes and albumin. Both ammonia detoxification performance and the secretion of albumin and enzymes were affected by the shape of the constructs, indicating that specific patterns influenced organoid functions. Additionally, the authors used iodixanol to optically tune the photocrosslinking of GelMA. Hydrogels containing iodixanol exhibited lower stiffness (2 kPa) compared to those without iodixanol (5 kPa), which is explained by the higher sol fraction of the GelMA/iodixanol hydrogels. When volumetrically bioprinted, organoids in GelMA grew larger than those in BME, GelMA (extrusion bioprinted), and GelMA (casted). An important consideration for GelMA hydrogels is their inherently elastic nature. Owing to the covalent crosslinking of the GelMA network, these matrices are almost purely elastic. In contrast, the liver exhibits viscoelastic behavior, and although several approaches have been developed to impart viscoelasticity to GelMA hydrogels, this aspect remains largely unexplored in the context of biliary organoids [125,126].

#### 3.2.3. Cellulose

Cellulose is an extremely abundant polysaccharide obtained from the cell wall of plants. Extraction of cellulose is achieved by oxidation mediated by 2,2,6,6-tetramethylpiperidinyl-1-oxyl (TEMPO), which allows the solubilization of the nanofibrils in water [127]. The cellulose nanofibrils (CNF), once dissolved, form hydrogels of which the stiffness can be tuned by changing their concentration [128,129]. Additionally, cellulose-based systems are suitable for 3D bioprinting as they are inherently shear thinning, highlighting their potential in regenerative medicine [129,130]. Krüger and colleagues investigated the use of cellulose nanofibril (CNF)-based hydrogels for the differentiation of biliary organoids into hepatocyte-like cells, employing relatively soft matrices (~255 Pa) with stiffness comparable to that of BME [74,131]. Interestingly, the hydrogels deformed up to 100% before breaking, and the hydrogel displayed a strain softening behavior. Other studies using lower cellulose concentrations (0.2% *w*/*v*) have demonstrated clear viscoelastic behavior in CNF hydrogels. Uniaxial load–unload compression tests revealed a pronounced hysteresis loop, indicative of time-dependent energy dissipation within the material, due to the hydrogen bonds that hold the fibrils together [132]. An important drawback of this system is that although it improves the hepatogenic differentiation compared to BME, the hydrogels do not support organoid growth and their morphological organization. In general, a major limitation of CNF hydrogels is that they lack cell adhesion sites, and the authors hypothesized that this is the reason for the lack of organoid proliferation. However, carbodiimide chemistry can be used to functionalize CNF with cell adhesion peptides, e.g., RGD. This approach has already been used to grow intestinal organoids, but to our knowledge there are no reports of biliary organoids cultured in cell-adhesive CNF hydrogels [133].

#### 3.2.4. Hyaluronan

Hyaluronan (hyaluronic acid, HA) is an anionic linear polysaccharide found in the ECM in most tissues and organs [134]. HA is scarcely present in the liver, but its content increases during fibrosis [135,136]. Per se, HA cannot form hydrogels. However, various strategies have been developed to obtain hydrogels with HA, primarily because of the biological relevance of HA in many tissues and its biological activity [137,138]. HA does not directly engage integrins, but functionalization of HA with RGD-bearing peptides is possible [139]. However, cells can still bind and recognize HA through CD44, RHAMM and ICAM-1, allowing for cell locomotion in the ECM [139]. Cell adhesion is still permitted by CD44, which is mostly expressed by mesenchymal and cancerous cells, and is implicated in mechanotransduction [140,141,142]. Importantly, CD44 is also expressed by liver progenitor cells and cholangiocytes, and HA is present in the liver [143,144]. In the context of biliary organoids, HA-based hydrogels were used by Rizwan et al. to study the role of viscoelasticity in driving tubulogenesis of cholangiocyte organoids through Notch signaling [145]. The authors engineered an HA oxime (HAO) matrix with tunable biophysical and biochemical properties, incorporated laminin, and crosslinked it using either an MMP-sensitive or an MMP-insensitive linkers. Laminin was crucial for the self-assembly of organoids, but it also shifted the nature of the system from almost purely elastic to viscoelastic (i.e., stress relaxing). However, by increasing the concentration of HAO the authors obtained slow relaxing matrices, which reduced both organoid growth and size. By comparing MMP-sensitive and MMP-insensitive linkers, the authors found that organoid growth was unaffected by matrix degradability. Additionally, the authors found that the fast-relaxing hydrogels, compared to the slow-relaxing ones, were upregulating classical YAP targets such as *Ctgf*, *Cyr61*, *Areg* and *Ankrd1*, confirming previous evidence that matrix viscoelasticity alone can induce YAP activation [88]. To determine the role of viscoelasticity in bile duct formation, the authors functionalized the HAO hydrogels by covalently binding Jag-1, the Notch-2 receptor ligand, to the network. It is already known that the Notch signaling pathway is critical in bile duct formation, but 3D culture system that achieved this usually required co-culture with stromal cells, which are known to stimulate Notch signaling in biliary organoids [42]. Interestingly, only the fast-relaxing hydrogels allowed bile duct formation, highlighting the critical role of viscoelasticity in biliary tissue organization. Overall, this study demonstrates that HA-based hydrogels, through tunable viscoelasticity and biochemical functionalization, provide a powerful platform to control cholangiocyte organoid growth, morphogenesis, and bile duct formation.

#### 3.2.5. Fibrin

Fibrinogen is a large glycoprotein that is cleaved off during the coagulation cascade and converted into insoluble fibrin monomers. The fibrin monomers then self-assemble into fibers of increasing length and diameter, forming fibrous hydrogels. These hydrogels are frequently used in tissue engineering thanks to their high biocompatibility, the numerous cell adhesion sites and the possibility of autologous production [146]. From a mechanical standpoint, fibrin hydrogels exhibit classical features of biological polymer matrices: relatively low stiffness, non-linear elasticity and viscoelastic behavior [90,92]. Broguiere et al. investigated the use of fibrin-based hydrogels for the culture of epithelial organoids [147]. The authors focused on developing a well-defined matrix that avoided the main disadvantages of BME. They used epithelial organoids including intestinal, biliary and pancreatic cells, and initially tested polyethylene glycol, alginate, hyaluronic acid, and fibrin hydrogels, mechanically tuned to match the stiffness of BME, either with or without 10% BME supplementation. No hydrogel, expect for the BME/fibrin one, allowed for organoid formation. Notably, the BME/fibrin hydrogel reached an organoid formation efficiency equal to that of BME, indicating that components from BME are crucial for organoid formation. Then, maintaining the BME supplementation, the stiffness of the fibrin hydrogels was modulated by changing the polymer concentration. Stiffnesses between ~80 to ~150 Pa were found optimal for organoid growth, while the softest ~30 Pa hydrogels did not form stable hydrogels and degraded. Instead, the stiffer hydrogels (~430 Pa) did not allow for organoid formation. By supplementing fibrin hydrogels with the main components of BME, the laminin-111/enactin complex (LEC) proved essential for sustaining organoid expansion, yielding proliferation levels comparable to those obtained in BME. Finally, human biliary, pancreatic and pancreatic cancer organoids were successfully cultured in the fibrin-laminin hydrogels, supporting their use in epithelial organoid culture. Biliary organoids cultured in the fibrin-laminin hydrogels correctly expressed the ductal CK19 and gene expression analysis showed no differences between biliary organoids cultured in fibrin-based hydrogels or BME. Altogether, these results elevate fibrin-laminin-based hydrogels as potential candidates to replace BME-based organoid systems, owing to their translational potential. One critical requirement missing, however, is the availability of clinical-grade laminin, as acknowledged by the authors. This shortcoming could be bypassed by incorporating peptides derived from laminin, mimicking its function in organoid formation and proliferation.

### 3.3. Synthetic Polymers for Hydrogel Fabrication

Synthetic polymers are formed by linking monomers through established polymerization reactions. They are widely used to fabricate hydrogels with mechanical properties that can be precisely tailored to experimental requirements [148]. However, synthetic polymers are usually inert and lack intrinsic bioactivity, but they can be engineered to introduce biologically active groups. By incorporating specific peptide sequences into their backbone, they can emulate extracellular matrix components, allowing for control over cell behavior.

#### 3.3.1. Polyisocyanopeptides

Polyisocyanopeptide (PIC) are used to fabricate highly tunable synthetic hydrogels. The polymer is formed with a core dipeptide, usually D and L alanine, that can be expanded to modulate the stiffness of the hydrogel [149]. Moreover, the length of the tail, consisting of ethylene glycol, can be varied to change the gelation temperature between 5 and 60 °C [150]. PIC hydrogels can be crosslinked by increasing the temperature in a fully revertible process, easing the harvesting of the cells. These hydrogels share characteristics of collagen and fibrin, as they have a fibrous architecture and, mechanically, possess strain hardening behavior. Both the stiffness and the strain hardening behavior of PIC hydrogels can be modulated by increasing the polymer length and/or increasing the polymer concentration. Notably, polymer concentration and porosity show an inverse correlation. However, changing the molecular weight of the polymer while maintaining the same concentration allows to fabricate hydrogels with different mechanical properties and constant porosity [151,152]. This allows PIC to be particularly useful for studies where a specific parameter must be isolated, as other polymer system often require increasing the polymer concentration, and hence to reduce the porosity, to increase stiffness. Finally, PIC can be functionalized with peptides to introduce specific cell adhesion ligands [153,154]. Ye et al. cultured biliary organoids in PIC hydrogels supplemented with laminin/entactin (LEC) to produce a synthetic, chemically defined and xeno-free alternative to BME to culture biliary organoids [155]. Similarly to what reported for fibrin-RGD hydrogels, the authors found that PIC-RGD hydrogels did not support the expansion of organoids. Instead, LEC addition improved organoid proliferation in a concentration dependent manner. Then, the authors employed different molecular weights and concentrations of PIC to tune the hydrogel stiffness. Interestingly, the softest (~18 Pa) hydrogels best supported organoid proliferation, while the stiffest (~83 Pa) hydrogels performed worse. Also, addition of LEC lowered the mechanical performance of the hydrogels, most likely due to sterical interference in network formation. Since the group effort was to produce a xeno-free alternative to BME, the authors replaced LEC with recombinant human laminin-111. The PIC-laminin hydrogels performed slightly worse compared to the PIC-LEC hydrogels but still succeeded in supporting the proliferation of biliary organoids. The same group recently studied PIC hydrogels for their potential to support biliary organoids differentiation into mature cholangiocytes, and compared them to BME/collagen and PIC/collagen hydrogels [156]. Additionally, the organoids were characterized functionally by testing the Farnesoid X receptor (FXR), which regulates bile acid excretion and prevents excessive bile acid accumulation and cholestasis. FXR activity was assessed by incubation with either an FXR agonist (GW4064) or with high concentrations of bile acids. In all hydrogel conditions, the organoids performed correct bile homeostasis. Finally, the authors employed PIC hydrogels to model the TGF-β–mediated fibrotic response. TGF-β stimulation induced a stronger fibrotic response in cholangiocyte organoids cultured within PIC matrices, as evidenced by increased expression of ACTA2, COL1A1, and TIMP1 relative to BME and PIC-collagen hydrogels. Overall, PIC hydrogels provide a tunable, xeno-free platform that supports biliary organoid growth and differentiation.

#### 3.3.2. Polyethylene Glycol

Polyethylene glycol (PEG) is a synthetic polymer commonly used in hydrogel fabrication. PEG is inert, biocompatible and provides an excellent backbone for numerous modifications. Indeed, different molecular geometries, e.g., multiarm, and molecular weights are readily available on the market [157,158]. Chemical modifications to introduce specific peptides, cell adhesion motifs, crosslinking strategies or functionalization with other molecules are possible [158,159,160]. Thanks to their flexibility, PEG hydrogels can be engineered to create elastic, viscoelastic, and viscoplastic matrices by varying the crosslinking dynamics in the network [161]. PEG-based matrices have been previously employed for organoid culture, usually by introducing cell adhesive peptides as well as MMP-sensitive sequences [158,162,163,164]. In our previous work, we developed a fully defined, mechano-modulatory 3D culture system based on PEG hydrogels to replace animal-derived matrices for biliary organoid culture [165]. We designed an enzymatically crosslinked PEG network functionalized with minimal adhesive motifs (RGD) and protease-sensitive peptides, allowing complete control over biochemical and biophysical parameters. By tuning the crosslinking density, we recreated a range of mechanical conditions spanning from the physiological stiffness of the healthy liver (~1.3 kPa) to that of fibrotic tissue (~4 kPa). We found that organoid formation was highly sensitive to matrix stiffness, with optimal growth occurring within the physiological range. Mechanistically, this mechano-responsiveness required activation of the integrin–Src–YAP axis, rather than acto-myosin contractility. Conversely, excessive stiffness—mimicking fibrotic conditions—reduced biliary organoid proliferation and induced a stress and inflammatory response. These findings demonstrated that matrix mechanics alone can elicit inflammation- and fibrosis-like transcriptional programs in biliary organoids. Importantly, this PEG system supported long-term expansion and differentiation of both mouse and human liver organoids, including those directly derived from patient biopsies, without any animal-derived components.

PEG can also serve as a structural component in fully synthetic hydrogels based on copolymeric networks. Wang et al. developed a tunable hydrogel composed of dendritic polyglycerol-bicyclononyne (dPG-BCN) and poly(N-isopropylacrylamide)-co-polyethylene glycol azide (pNIPAAm-co-PEG-N3) [166]. This hydrogel served as a platform to culture human iPSC-derived hepatoblast organoids and to control their hepatic lineage specification. By systematically varying hydrogel stiffness, the authors identified ~400 Pa as the optimal value to sustain organoid formation and growth, whereas softer (~270 Pa) or stiffer (~500, ~700, and ~1400 Pa) matrices limited organoid expansion. Moreover, integrin-mediated TGF-β signaling proved crucial in directing hepatic fate: RGD-functionalized hydrogels enhanced integrin-dependent TGF-β activation and promoted cholangiocyte differentiation, while RGD-free matrices favored hepatocyte commitment. This work demonstrated how matrix design can precisely regulate iPSC lineage specification through hydrogel engineering, providing a fully synthetic, xeno-free alternative to BME for liver organoid generation.

The synthetic and FDA-approved nature of PEG provides a translational advantage, offering a reproducible and clinically compatible platform for ex vivo tissue culture. In this context, combining the synthetic hydrogel approach with the regenerative capacity of cholangiocyte organoids described by Sampaziotis et al. could pave the way for engineering fully defined, transplantable biliary grafts optimized for mechanical compatibility and functional integration within the native bile duct [65].

## 4. Discussion and Future Perspectives

**Figure 3:**
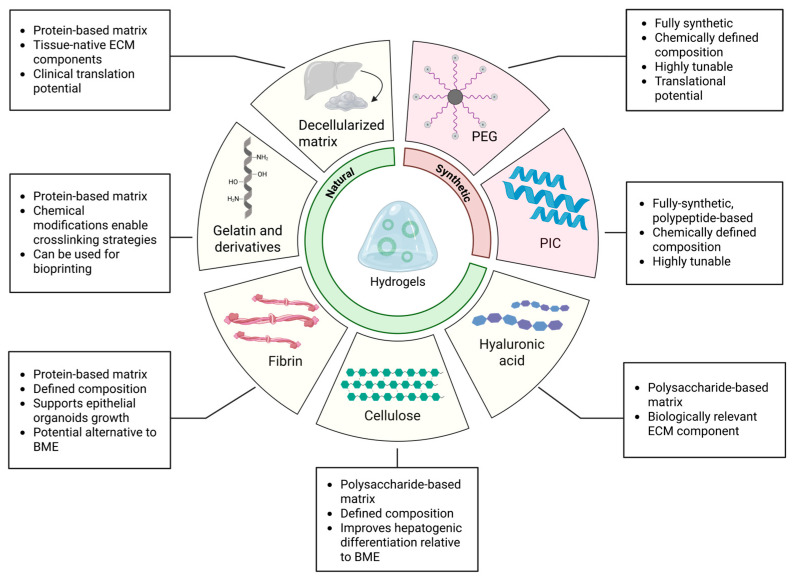
Natural and semi-synthetic polymers for hydrogel fabrication. Schematic overview of hydrogel systems employed for biliary organoid bioengineering. Natural polymers—including decellularized liver matrix, gelatin derivatives, fibrin, cellulose, and hyaluronic acid—offer intrinsic biocompatibility and bioactivity but limited tunability. Synthetic polymers such as polyethylene glycol (PEG) and polyisocyanopeptides (PIC) provide chemically defined, modular platforms that enable precise control over mechanical and biochemical cues. Together, these matrices constitute a versatile toolkit for modeling biliary tissue physiology, disease, and regeneration in vitro. Created in BioRender. Sorrentino, G. (2025) https://BioRender.com/9ym0xl3 (accessed on 14 November 2025).

Three-dimensional culture systems for biliary organoids have historically relied on tumor-derived matrices such as BME. While BME has been instrumental in enabling organoid formation, its undefined composition, xenogeneic origin, and high batch-to-batch variability pose major limitations for reproducibility, mechanistic studies, and clinical translation. In response, next-generation hydrogels—derived from natural or synthetic sources—have emerged as powerful alternatives for engineering more controlled and physiologically relevant microenvironments (Figure 3, Table 1).

Tissue-specific hydrogels, such as those based on decellularized liver extracellular matrix, have demonstrated the ability to support organoid proliferation and differentiation while closely mimicking the native niche [111,117]. In parallel, synthetic hydrogels composed of clinically relevant materials like PEG and PIC enable robust expansion of human biliary organoids under defined and tunable conditions and recapitulate the mechanical properties of healthy or fibrotic liver tissue, inducing disease-relevant phenotypes [155,156,165]. Such platforms not only improve reproducibility but also facilitate mechanistic investigations of liver pathophysiology. In addition, semi-synthetic hydrogels that combine the bioactivity of natural polymers with the mechanical tunability of synthetic materials offer a balanced solution to overcome the limitations of purely synthetic systems. These matrices enhance cell–matrix interactions while providing sufficient control over structural and mechanical parameters, improving the physiological relevance of in vitro organoid cultures.

Despite these promising advances, there is still no consensus on the optimal biomechanical and biochemical parameters required for biliary organoid development and maturation. For example, most studies have primarily focused on static stiffness, often overlooking more dynamic matrix properties such as viscoelasticity and plasticity, which are critical regulators of tissue morphogenesis and cell behavior in vivo. A deeper understanding of how time-dependent mechanical cues influence organoid morphogenesis and function is urgently needed. Recent evidence suggests that fast-relaxing viscoelastic matrices can enhance organoid expansion and tubulogenesis by modulating mechanotransductive pathways like YAP signaling, even in the absence of matrix degradation [145]. Moreover, functionalizing hydrogels with immobilized biochemical ligands such as Jagged-1 can synergize with mechanical cues to drive bile duct-like architecture through activation of pathways such as Notch. These findings highlight the potential of engineering hydrogels that recapitulate not just the structural, but also the dynamic mechanical properties of the liver microenvironment.

In future, a central goal will be the development of smart, tunable hydrogels that can dynamically adjust their stiffness, crosslinking density, and biochemical signaling in response to external stimuli or endogenous cellular activity [167]. This level of spatiotemporal control will be critical to more precisely direct organoid differentiation or disease phenotypes, enabling researchers to mimic developmental transitions and pathological cues with higher fidelity. Such responsive materials must be biocompatible and composed of clinically approved components, xeno-free components and produced through standardized processes that minimize batch variability to facilitate their integration into translational workflows. Injectable hydrogels that promote organoid delivery, engraftment, and integration into host tissue will be indispensable for advancing regenerative medicine applications; however, a major challenge will be matching their degradation rate with tissue remodeling and biliary tree formation. Preclinical studies have already demonstrated the capacity of biliary organoids to engraft and restore ductal function in injured bile ducts; translating these findings into clinical therapies will require supportive matrices that combine mechanical integrity with biodegradability and immune compatibility [168].

Beyond regenerative applications, next-generation hydrogels will unlock new possibilities for modeling liver and, in particular, biliary diseases in vitro. By adjusting matrix stiffness and biochemical composition, researchers can simulate fibrotic or tumorigenic environments, thereby inducing disease-like phenotypes in biliary organoids. Organoids embedded in fibrosis mimicking hydrogels or exposed to oncogenic cues have already shown the capacity to mirror key pathological features, providing a platform for mechanistic studies and drug screening. Patient-derived organoids combined with disease-tailored hydrogels will also advance personalized approaches to studying rare or complex biliary disorders.

To increase physiological relevance, integrating biliary or tumor-derived organoids with stromal and immune cell types in co-culture systems embedded within hydrogels will be essential. These multicellular platforms will allow the investigation of cell–cell communication, tumor–stroma crosstalk, and immune modulation, providing deeper insights into disease mechanisms and therapeutic responses. Additionally, combining these organoid systems with enabling technologies such as bioprinting, microfluidics, and biosensing will pave the way for more sophisticated and predictive disease models. Bioprinted bile duct constructs, organ-on-chip systems simulating bile flow and interstitial pressure, and platforms with real-time monitoring of luminal conditions represent key frontiers in this evolving field.

In conclusion, biliary organoids stand at the convergence of stem cell biology, biomaterials science, and translational medicine. Their successful application in research and therapy hinges on interdisciplinary collaboration that integrates expertise in material engineering, mechanobiology, liver development, and clinical hepatology. By advancing hydrogel engineering and standardizing organoid culture systems, the field is poised to deliver robust, functional, and clinically applicable models that will transform our understanding and treatment of biliary diseases.

## Figures and Tables

**Table 1:** Main properties of the hydrogels employed for biliary organoid engineering.

Hydrogel	Composition	Mechanical Profile	Application	Pros	Cons	Reference
**Decellularized ECM (dECM)**	Decellularized liver ECM	Stiffness: ~250 Pa (human liver), ~650 Pa (porcine liver) Viscoelastic	Organoid culture and differentiation	Tissue-specific ECM; supports organoid growth and differentiation; ECM components are preserved; possibility of clinical applications	Requires modification to tune mechanical properties; relies on tissue availability	[111,117]
	Decellularized CCA ECM	Stiffness: n/a^1^ Viscoelastic	Organoid culture	Cancer-specific ECM; CCA patient-derived organoids contribute to desmoplasia in non-tumoral dECM hydrogels		
**GelMA, GelSH**	Hydrolyzed collagen–chemically modified	Stiffness: 2.0–9.6 kPa (GelMA, tunable); 6.2–32.5 kPa (GelSH, depending on modification) Elastic	Organoid culture and differentiation; organoid volumetric bioprinting	Cell adhesive; suitable for hepatogenic differentiation; stiffness can be tuned to support organoid growth	Gelatin requires modification to form mechanically stable hydrogels at low concentrations; elastic (strategies to make viscoelastic hydrogels are possible)	[120,123,124]
**Cellulose**	TEMPO-oxidized cellulose nanofibrils	Stiffness: ~255 Pa Viscoelastic	Organoid differentiation	Widely available; improves hepatogenic differentiation of biliary organoids	Low mechanical properties; doesn’t allow for organoid growth	[131]
**Hyaluronan (HA)**	HA functionalized with RGD peptides and laminin	Stiffness: 0.2–1.0 kPa Viscoelastic (tunable with laminin addition)	Organoid culture	Mechanical properties can be independently tuned	Does not possess integrin-binding sequences	[145]
**Fibrin**	Fibrin supplemented with laminin	Stiffness: 30–430 Pa Viscoelastic	Organoid culture	Defined composition; stiffness can be tuned to support optimal organoid growth	Requires laminin to support organoid proliferation	[147]
**Polyisocyanopeptides (PIC)**	Fully synthetic; peptide-based polymer; functionalized with cell adhesive sequences	Stiffness: ~18–~83 Pa; Non-linear elastic	Organoid culture and differentiation	Chemically defined; xeno-free; stiffness can be tuned to support optimal organoid growth	Requires modification to introduce cell-adhesive ligands	[155,156]
**Polyethylene glycol (PEG)**	Fully synthetic; functionalized with cell adhesive sequences	1.3–4.0 kPa (PEG); Elastic	Organoid culture and differentiation	Chemically defined; xeno-free; support long-term culture and hepatogenic differentiation; stiffness can be tuned to support optimal organoid growth	Elastic (strategies to produce viscoelastic and viscoplastic hydrogels are available); requires modification to introduce cell-adhesive ligands	[165,166]
		270 Pa–1.4 kPa (pNIPAAm-co-PEG-N3/dPG-BCN) Viscoelastic	Organoid culture and differentiation	Hydrogel design allows to control hepatoblasts differentiation; stiffness can be tuned to support optimal organoid growth	Requires modification to introduce cell-adhesive ligands	

^1^ not available.

## Data Availability

No new data were created or analyzed in this study.

## References

[1] Roskams T., Desmet V. (2008). Embryology of Extra- and Intrahepatic Bile Ducts, the Ductal Plate. Anat. Rec..

[2] Boyer J.L. (2013). Bile Formation and Secretion. Compr. Physiol..

[3] Cardinale V., Wang Y., Carpino G., Mendel G., Alpini G., Gaudio E., Reid L.M., Alvaro D. (2012). The Biliary Tree-a Reservoir of Multipotent Stem Cells. Nat. Rev. Gastroenterol. Hepatol..

[4] Spence J.R., Lange A.W., Lin S.C.J., Kaestner K.H., Lowy A.M., Kim I., Whitsett J.A., Wells J.M. (2009). Sox17 Regulates Organ Lineage Segregation of Ventral Foregut Progenitor Cells. Dev. Cell.

[5] Lou C., Lan T., Xu S., Hu X., Li J., Xiang Z., Lin S., Fan X., Chen J., Xu X. (2025). Heterogeneity and Plasticity of Cholangiocytes in Liver Injury: A Journey from Pathophysiology to Therapeutic Utility. Gut.

[6] So J., Kim A., Lee S.H., Shin D. (2020). Liver Progenitor Cell-Driven Liver Regeneration. Exp. Mol. Med..

[7] Tarlow B.D., Finegold M.J., Grompe M. (2014). Clonal Tracing of Sox9+ Liver Progenitors in Mouse Oval Cell Injury. Hepatology.

[8] Fausto N., Campbell J.S. (2003). The Role of Hepatocytes and Oval Cells in Liver Regeneration and Repopulation. Mech. Dev..

[9] Tarlow B.D., Pelz C., Naugler W.E., Wakefield L., Wilson E.M., Finegold M.J., Grompe M. (2014). Bipotential Adult Liver Progenitors Are Derived from Chronically Injured Mature Hepatocytes. Cell Stem Cell.

[10] Lazaridis K.N., Larusso N.F. (2015). The Cholangiopathies. Mayo Clin. Proc..

[11] Squires R.H., Ng V., Romero R., Ekong U., Hardikar W., Emre S., Mazariegos G.V. (2014). Evaluation of the Pediatric Patient for Liver Transplantation: 2014 Practice Guideline by the American Association for the Study of Liver Diseases, American Society of Transplantation and the North American Society for Pediatric Gastroenterology, Hepatolo. Hepatology.

[12] Tam P.K.H., Yiu R.S., Lendahl U., Andersson E.R. (2018). Cholangiopathies—Towards a Molecular Understanding. EBioMedicine.

[13] Sorrentino G. (2025). Microenvironmental Control of the Ductular Reaction: Balancing Repair and Disease Progression. Cell Death Dis..

[14] Sato K., Marzioni M., Meng F., Francis H., Glaser S., Alpini G. (2019). Ductular Reaction in Liver Diseases: Pathological Mechanisms and Translational Significances. Hepatology.

[15] Fabris L., Cadamuro M., Guido M., Spirli C., Fiorotto R., Colledan M., Torre G., Alberti D., Sonzogni A., Okolicsanyi L. (2007). Analysis of Liver Repair Mechanisms in Alagille Syndrome and Biliary Atresia Reveals a Role for Notch Signaling. Am. J. Pathol..

[16] Chung B.K., Karlsen T.H., Folseraas T. (2018). Cholangiocytes in the Pathogenesis of Primary Sclerosing Cholangitis and Development of Cholangiocarcinoma.

[17] Guicciardi M.E., Trussoni C.E., LaRusso N.F., Gores G.J. (2020). The Spectrum of Reactive Cholangiocytes in Primary Sclerosing Cholangitis. Hepatology.

[18] Jalan-Sakrikar N., Guicciardi M.E., O’Hara S.P., Azad A., LaRusso N.F., Gores G.J., Huebert R.C. (2024). Central Role for Cholangiocyte Pathobiology in Cholestatic Liver Diseases. Hepatology.

[19] Baiocchini A., Montaldo C., Conigliaro A., Grimaldi A., Correani V., Mura F., Ciccosanti F., Rotiroti N., Brenna A., Montalbano M. (2016). Extracellular Matrix Molecular Remodeling in Human Liver Fibrosis Evolution. PLoS ONE.

[20] Greenman R., Segal-Salto M., Barashi N., Hay O., Katav A., Levi O., Vaknin I., Aricha R., Aharoni S., Snir T. (2023). CCL24 Regulates Biliary Inflammation and Fibrosis in Primary Sclerosing Cholangitis. JCI Insight.

[21] Klaas M., Kangur T., Viil J., Mäemets-Allas K., Minajeva A., Vadi K., Antsov M., Lapidus N., Järvekülg M., Jaks V. (2016). The Alterations in the Extracellular Matrix Composition Guide the Repair of Damaged Liver Tissue. Sci. Rep..

[22] Manns M.P., Bergquist A., Karlsen T.H., Levy C., Muir A.J., Ponsioen C., Trauner M., Wong G., Younossi Z.M. (2025). Primary Sclerosing Cholangitis. Nat. Rev. Dis. Primers.

[23] Lakshminarayanan B., Davenport M. (2016). Biliary Atresia: A Comprehensive Review. J. Autoimmun..

[24] Tam P.K.H., Wells R.G., Tang C.S.M., Lui V.C.H., Hukkinen M., Luque C.D., De Coppi P., Mack C.L., Pakarinen M., Davenport M. (2024). Biliary Atresia. Nat. Rev. Dis. Primers.

[25] Gilbert M.A., Spinner N.B. (2017). Alagille Syndrome: Genetics and Functional Models. Curr. Pathobiol. Rep..

[26] Li L., Krantz I.D., Deng Y., Genin A., Banta A.B., Collins C.C., Qi M., Trask B.J., Kuo W.L., Cochran J. (1997). Alagille Syndrome Is Caused by Mutations in Human Jagged1, Which Encodes a Ligand for Notch1. Nat. Genet..

[27] Warthen D.M., Moore E.C., Kamath B.M., Morrissette J.J.D., Sanchez P., Piccoli D.A., Krantz I.D., Spinner N.B. (2006). Jagged1 (JAG1) Mutations in Alagille Syndrome: Increasing the Mutation Detection Rate. Hum. Mutat..

[28] Ooi C.Y., Durie P.R. (2016). Cystic Fibrosis from the Gastroenterologist’s Perspective. Nat. Rev. Gastroenterol. Hepatol..

[29] Olaizola P., Rodrigues P.M., Caballero-Camino F.J., Izquierdo-Sanchez L., Aspichueta P., Bujanda L., Larusso N.F., Drenth J.P.H., Perugorria M.J., Banales J.M. (2022). Genetics, Pathobiology and Therapeutic Opportunities of Polycystic Liver Disease. Nat. Rev. Gastroenterol. Hepatol..

[30] Banales J.M., Marin J.J.G., Lamarca A., Rodrigues P.M., Khan S.A., Roberts L.R., Cardinale V., Carpino G., Andersen J.B., Braconi C. (2020). Cholangiocarcinoma 2020: The next Horizon in Mechanisms and Management. Nat. Rev. Gastroenterol. Hepatol..

[31] Khan A.S., Dageforde L.A. (2019). Cholangiocarcinoma. Surg. Clin. N. Am..

[32] Cai X., Zhai J., Kaplan D.E., Zhang Y., Zhou L., Chen X., Qian G., Zhao Q., Li Y., Gao L. (2012). Background Progenitor Activation Is Associated with Recurrence after Hepatectomy of Combined Hepatocellular-Cholangiocarcinoma. Hepatology.

[33] Komuta M., Spee B., Borght S.V., De Vos R., Verslype C., Aerts R., Yano H., Suzuki T., Matsuda M., Fujii H. (2008). Clinicopathological Study on Cholangiolocellular Carcinoma Suggesting Hepatic Progenitor Cell Origin. Hepatology.

[34] Petrick J.L., Yang B., Altekruse S.F., Van Dyke A.L., Koshiol J., Graubard B.I., McGlynn K.A. (2017). Risk Factors for Intrahepatic and Extrahepatic Cholangiocarcinoma in the United States: A Population-Based Study in SEER-Medicare. PLoS ONE.

[35] Sirica A.E., Strazzabosco M., Cadamuro M. (2021). Intrahepatic Cholangiocarcinoma: Morpho-Molecular Pathology, Tumor Reactive Microenvironment, and Malignant Progression. Adv. Cancer Res..

[36] Cadamuro M., Stecca T., Brivio S., Mariotti V., Fiorotto R., Spirli C., Strazzabosco M., Fabris L. (2018). The Deleterious Interplay between Tumor Epithelia and Stroma in Cholangiocarcinoma. Biochim. Biophys. Acta Mol. Basis Dis..

[37] Sirica A.E., Gores G.J. (2014). Desmoplastic Stroma and Cholangiocarcinoma: Clinical Implications and Therapeutic Targeting. Hepatology.

[38] Marsee A., Roos F.J.M., Verstegen M.M.A., Roos F.J.M., Verstegen M.M.A., Clevers H., Vallier L., Takebe T., Huch M., Peng W.C. (2021). Building Consensus on Definition and Nomenclature of Hepatic, Pancreatic, and Biliary Organoids. Cell Stem Cell.

[39] Dianat N., Dubois-Pot-Schneider H., Steichen C., Desterke C., Leclerc P., Raveux A., Combettes L., Weber A., Corlu A., Dubart-Kupperschmitt A. (2014). Generation of Functional Cholangiocyte-like Cells from Human Pluripotent Stem Cells and HepaRG Cells. Hepatology.

[40] Ramli M.N.B., Lim Y.S., Koe C.T., Demircioglu D., Tng W., Gonzales K.A.U., Tan C.P., Szczerbinska I., Liang H., Soe E.L. (2020). Human Pluripotent Stem Cell-Derived Organoids as Models of Liver Disease. Gastroenterology.

[41] Sampaziotis F., De Brito M.C., Madrigal P., Bertero A., Saeb-Parsy K., Soares F.A.C., Schrumpf E., Melum E., Karlsen T.H., Bradley J.A. (2015). Cholangiocytes Derived from Human Induced Pluripotent Stem Cells for Disease Modeling and Drug Validation. Nat. Biotechnol..

[42] Ogawa M., Ogawa S., Bear C.E., Ahmadi S., Chin S., Li B., Grompe M., Keller G., Kamath B.M., Ghanekar A. (2015). Directed Differentiation of Cholangiocytes from Human Pluripotent Stem Cells. Nat. Biotechnol..

[43] Ogawa M., Jiang J.X., Xia S., Yang D., Ding A., Laselva O., Hernandez M., Cui C., Higuchi Y., Suemizu H. (2021). Generation of Functional Ciliated Cholangiocytes from Human Pluripotent Stem Cells. Nat. Commun..

[44] Soroka C.J., Assis D.N., Boyer J.L. (2019). Patient-Derived Organoids from Human Bile: An in Vitro Method to Study Cholangiopathies. Methods in Molecular Biology.

[45] Huch M., Dorrell C., Boj S.F., Van Es J.H., Li V.S.W., Van De Wetering M., Sato T., Hamer K., Sasaki N., Finegold M.J. (2013). In Vitro Expansion of Single Lgr5 + Liver Stem Cells Induced by Wnt-Driven Regeneration. Nature.

[46] Huch M., Gehart H., Van Boxtel R., Hamer K., Blokzijl F., Verstegen M.M.A., Ellis E., Van Wenum M., Fuchs S.A., De Ligt J. (2015). Long-Term Culture of Genome-Stable Bipotent Stem Cells from Adult Human Liver. Cell.

[47] Broutier L., Andersson-Rolf A., Hindley C.J., Boj S.F., Clevers H., Koo B.K., Huch M. (2016). Culture and Establishment of Self-Renewing Human and Mouse Adult Liver and Pancreas 3D Organoids and Their Genetic Manipulation. Nat. Protoc..

[48] Chen C., Jochems P.G.M., Salz L., Schneeberger K., Penning L.C., Van De Graaf S.F.J., Beuers U., Clevers H., Geijsen N., Masereeuw R. (2018). Bioengineered Bile Ducts Recapitulate Key Cholangiocyte Functions. Biofabrication.

[49] Nguyen R., Da Won Bae S., Qiao L., George J. (2021). Developing Liver Organoids from Induced Pluripotent Stem Cells (IPSCs): An Alternative Source of Organoid Generation for Liver Cancer Research. Cancer Lett..

[50] Si-Tayeb K., Lemaigre F.P., Duncan S.A. (2010). Organogenesis and Development of the Liver. Dev. Cell.

[51] Soroka C.J., Assis D.N., Alrabadi L.S., Roberts S., Cusack L., Jaffe A.B., Boyer J.L. (2019). Bile-Derived Organoids From Patients With Primary Sclerosing Cholangitis Recapitulate Their Inflammatory Immune Profile. Hepatology.

[52] Babu R.O., Lui V.C.H., Chen Y., Yiu R.S.W., Ye Y., Niu B., Wu Z., Zhang R., Yu M.O.N., Chung P.H.Y. (2020). Beta-Amyloid Deposition around Hepatic Bile Ducts Is a Novel Pathobiological and Diagnostic Feature of Biliary Atresia. J. Hepatol..

[53] Bijvelds M.J.C., Roos F.J.M., Meijsen K.F., Roest H.P., Verstegen M.M.A., Janssens H.M., van der Laan L.J.W., de Jonge H.R. (2022). Rescue of Chloride and Bicarbonate Transport by Elexacaftor-Ivacaftor-Tezacaftor in Organoid-Derived CF Intestinal and Cholangiocyte Monolayers. J. Cyst. Fibros..

[54] Waisbourd-Zinman O., Koh H., Tsai S., Lavrut P.M., Dang C., Zhao X., Pack M., Cave J., Hawes M., Koo K.A. (2016). The Toxin Biliatresone Causes Mouse Extrahepatic Cholangiocyte Damage and Fibrosis through Decreased Glutathione and SOX17. Hepatology.

[55] Fried S., Gilboa D., Har-Zahav A., Lavrut P.M., Du Y., Karjoo S., Russo P., Shamir R., Wells R.G., Waisbourd-Zinman O. (2020). Extrahepatic Cholangiocyte Obstruction Is Mediated by Decreased Glutathione, Wnt and Notch Signaling Pathways in a Toxic Model of Biliary Atresia. Sci. Rep..

[56] Andersson E.R., Chivukula I.V., Hankeova S., Sjöqvist M., Tsoi Y.L., Ramsköld D., Masek J., Elmansuri A., Hoogendoorn A., Vazquez E. (2018). Mouse Model of Alagille Syndrome and Mechanisms of Jagged1 Missense Mutations. Gastroenterology.

[57] Iqbal A., Van Hul N., Belicova L., Corbat A.A., Hankeova S., Andersson E.R., Van Hul N., Belicova L., Corbat A.A., Hankeova S. (2024). Spatially Segregated Defects and IGF1-Responsiveness of Hilar and Peripheral Biliary Organoids from a Model of Alagille Syndrome. Liver Int..

[58] Gribben C., Galanakis V., Calderwood A., Williams E.C., Chazarra-Gil R., Larraz M., Frau C., Puengel T., Guillot A., Rouhani F.J. (2024). Acquisition of Epithelial Plasticity in Human Chronic Liver Disease. Nature.

[59] Nuciforo S., Fofana I., Matter M.S., Blumer T., Calabrese D., Boldanova T., Piscuoglio S., Wieland S., Ringnalda F., Schwank G. (2018). Organoid Models of Human Liver Cancers Derived from Tumor Needle Biopsies. Cell Rep..

[60] Broutier L., Mastrogiovanni G., Verstegen M.M.A., Francies H.E., Gavarró L.M., Bradshaw C.R., Allen G.E., Arnes-Benito R., Sidorova O., Gaspersz M.P. (2017). Human Primary Liver Cancer-Derived Organoid Cultures for Disease Modeling and Drug Screening. Nat. Med..

[61] Li L., Knutsdottir H., Hui K., Weiss M.J., He J., Philosophe B., Cameron A.M., Wolfgang C.L., Pawlik T.M., Ghiaur G. (2019). Human Primary Liver Cancer Organoids Reveal Intratumor and Interpatient Drug Response Heterogeneity. JCI Insight.

[62] Fujiwara H., Tateishi K., Misumi K., Hayashi A., Igarashi K., Kato H., Nakatsuka T., Suzuki N., Yamamoto K., Kudo Y. (2019). Mutant IDH1 Confers Resistance to Energy Stress in Normal Biliary Cells through PFKP-Induced Aerobic Glycolysis and AMPK Activation. Sci. Rep..

[63] Cristinziano G., Porru M., Lamberti D., Buglioni S., Rollo F., Amoreo C.A., Manni I., Giannarelli D., Cristofoletti C., Russo G. (2021). FGFR2 Fusion Proteins Drive Oncogenic Transformation of Mouse Liver Organoids towards Cholangiocarcinoma. J. Hepatol..

[64] Roos F.J.M., van Tienderen G.S., Wu H., Bordeu I., Vinke D., Albarinos L.M., Monfils K., Niesten S., Smits R., Willemse J. (2022). Human Branching Cholangiocyte Organoids Recapitulate Functional Bile Duct Formation. Cell Stem Cell.

[65] Sampaziotis F., Muraro D., Tysoe O.C., Sawiak S., Beach T.E., Godfrey E.M., Upponi S.S., Brevini T., Wesley B.T., Garcia-Bernardo J. (2021). Cholangiocyte Organoids Can Repair Bile Ducts after Transplantation in the Human Liver. Science.

[66] Zhu Y., Yang W., Wang Z., Chen D., Wang J., Ren H. (2025). Constructing Biomimetic Microenvironments for Liver Regeneration. J. Nanobiotechnology.

[67] Elci B.S., Nikolaev M., Rezakhani S., Lutolf M.P. (2024). Bioengineered Tubular Biliary Organoids. Adv. Heal. Healthc. Mater..

[68] Jin Y., Kim J., Lee J.S., Min S., Kim S., Ahn D.H., Kim Y.G., Cho S.W. (2018). Vascularized Liver Organoids Generated Using Induced Hepatic Tissue and Dynamic Liver-Specific Microenvironment as a Drug Testing Platform. Adv. Funct. Mater..

[69] Lin X., Li J., Wang J., Filppula A.M., Zhang H., Zhao Y. (2024). Ion-Specific Hydrogel Microcarriers with Biomimetic Niches for Bioartifical Liver System. Adv. Funct. Mater..

[70] Kleinman H.K., Martin G.R. (2005). Matrigel: Basement Membrane Matrix with Biological Activity. Semin. Cancer Biol..

[71] Aisenbrey E.A., Murphy W.L. (2020). Synthetic Alternatives to Matrigel. Nat. Rev. Mater..

[72] Hughes C.S., Postovit L.M., Lajoie G.A. (2010). Matrigel: A Complex Protein Mixture Required for Optimal Growth of Cell Culture. Proteomics.

[73] Reed J., Walczak W.J., Petzold O.N., Gimzewski J.K. (2009). In Situ Mechanical Interferometry of Matrigel Films. Langmuir.

[74] Soofi S.S., Last J.A., Liliensiek S.J., Nealey P.F., Murphy C.J. (2009). The Elastic Modulus of Matrigel^TM^ as Determined by Atomic Force Microscopy. J. Struct. Biol..

[75] Chaudhuri O., Cooper-White J., Janmey P.A., Mooney D.J., Shenoy V.B. (2020). Effects of Extracellular Matrix Viscoelasticity on Cellular Behaviour.

[76] Chaudhuri O., Koshy S.T., Branco Da Cunha C., Shin J.W., Verbeke C.S., Allison K.H., Mooney D.J. (2014). Extracellular Matrix Stiffness and Composition Jointly Regulate the Induction of Malignant Phenotypes in Mammary Epithelium. Nat. Mater..

[77] Kumar P., Smith T., Raeman R., Chopyk D.M., Brink H., Liu Y., Sulchek T., Anania F.A. (2018). Periostin Promotes Liver Fibrogenesis by Activating Lysyl Oxidase in Hepatic Stellate Cells. J. Biol. Chem..

[78] Yin M., Woollard J., Wang X., Torres V.E., Harris P.C., Ward C.J., Glaser K.J., Manduca A., Ehman R.L. (2007). Quantitative Assessment of Hepatic Fibrosis in an Animal Model with Magnetic Resonance Elastography. Magn. Reson. Med..

[79] Fan W., Adebowale K., Váncza L., Li Y.Y., Rabbi M.F., Kunimoto K., Chen D., Mozes G., Chiu D.K.C., Li Y.Y. (2024). Matrix Viscoelasticity Promotes Liver Cancer Progression in the Pre-Cirrhotic Liver. Nature.

[80] Schrader J., Gordon-Walker T.T., Aucott R.L., van Deemter M., Quaas A., Walsh S., Benten D., Forbes S.J., Wells R.G., Iredale J.P. (2011). Matrix Stiffness Modulates Proliferation, Chemotherapeutic Response, and Dormancy in Hepatocellular Carcinoma Cells. Hepatology.

[81] Georges P.C., Hui J.J., Gombos Z., McCormick M.E., Wang A.Y., Uemura M., Mick R., Janmey P.A., Furth E.E., Wells R.G. (2007). Increased Stiffness of the Rat Liver Precedes Matrix Deposition: Implications for Fibrosis. Am. J. Physiol. Gastrointest. Liver Physiol..

[82] Wells R.G. (2008). The Role of Matrix Stiffness in Regulating Cell Behavior. Hepatology.

[83] Kozlowski M.T., Crook C.J., Ku H.T. (2021). Towards Organoid Culture without Matrigel. Commun. Biol..

[84] Drury J.L., Mooney D.J. (2003). Hydrogels for Tissue Engineering: Scaffold Design Variables and Applications. Biomaterials.

[85] Aswathy S.H., Narendrakumar U., Manjubala I. (2020). Commercial Hydrogels for Biomedical Applications. Heliyon.

[86] Ahmed S., Alshehri E., Nazneen S., Attia F., Obeid D., Almuzaini H., Alzahrani A., Salma J., Fujitsuka I., Assiri A.M. (2025). Current Advances and Prospects in Biomaterials-Guided Tools for Liver Organoids Research. Eng. Regen..

[87] Zhao J., Zhi Y., Ren H., Wang J., Zhao Y. (2025). Emerging Biotechnologies for Engineering Liver Organoids. Bioact. Mater..

[88] Elosegui-Artola A., Gupta A., Najibi A.J., Seo B.R., Garry R., Tringides C.M., de Lázaro I., Darnell M., Gu W., Zhou Q. (2023). Matrix Viscoelasticity Controls Spatiotemporal Tissue Organization. Nat. Mater..

[89] Bonakdar N., Gerum R., Kuhn M., Spörrer M., Lippert A., Schneider W., Aifantis K.E., Fabry B. (2016). Mechanical Plasticity of Cells. Nat. Mater..

[90] Erk K.A., Henderson K.J., Shull K.R. (2010). Strain Stiffening in Synthetic and Biopolymer Networks. Biomacromolecules.

[91] Shen Z.L., Dodge M.R., Kahn H., Ballarini R., Eppell S.J. (2008). Stress-Strain Experiments on Individual Collagen Fibrils. Biophys. J..

[92] Nam S., Hu K.H., Butte M.J., Chaudhuri O. (2016). Strain-Enhanced Stress Relaxation Impacts Nonlinear Elasticity in Collagen Gels. Proc. Natl. Acad. Sci. USA.

[93] Gardel M.L., Shin J.H., MacKintosh F.C., Mahadevan L., Matsudaira P., Weitz D.A. (2004). Elastic Behavior of Cross-Linked and Bundled Actin Networks. Science.

[94] Storm C., Pastore J.J., MacKintosh F.C., Lubensky T.C., Janmey P.A. (2005). Nonlinear Elasticity in Biological Gels. Nature.

[95] Duarte L.K.R., Rizzi L.G. (2024). Revisiting the Strain-Induced Softening Behaviour in Hydrogels. Soft Matter.

[96] Wisdom K.M., Adebowale K., Chang J., Lee J.Y., Nam S., Desai R., Rossen N.S., Rafat M., West R.B., Hodgson L. (2018). Matrix Mechanical Plasticity Regulates Cancer Cell Migration through Confining Microenvironments. Nat. Commun..

[97] Nam S., Lee J., Brownfield D.G., Chaudhuri O. (2016). Viscoplasticity Enables Mechanical Remodeling of Matrix by Cells. Biophys. J..

[98] Bertsch P., Sacco P. (2025). The Role of Non-Linear Viscoelastic Hydrogel Mechanics in Cell Culture and Transduction. Mater. Today Bio..

[99] Parada G.A., Zhao X. (2018). Ideal Reversible Polymer Networks. Soft Matter.

[100] Adebowale K., Allan C., Ha B., Saraswathibhatla A., Zhu J., Indana D., Popescu M.C., Demirdjian S., Martinez H.A., Esclamado A. (2025). Monocytes Use Protrusive Forces to Generate Migration Paths in Viscoelastic Collagen-Based Extracellular Matrices. Proc. Natl. Acad. Sci. USA.

[101] Wu Y., Song Y., Soto J., Hoffman T., Lin X., Zhang A., Chen S., Massad R.N., Han X., Qi D. (2025). Viscoelastic Extracellular Matrix Enhances Epigenetic Remodeling and Cellular Plasticity. Nat. Commun..

[102] Adu-Berchie K., Liu Y., Zhang D.K.Y., Freedman B.R., Brockman J.M., Vining K.H., Nerger B.A., Garmilla A., Mooney D.J. (2023). Generation of Functionally Distinct T-Cell Populations by Altering the Viscoelasticity of Their Extracellular Matrix. Nat. Biomed. Eng..

[103] Zwirner J., Devananthan P., Kabaliuk N., Docherty P.D., Ondruschka B. (2025). The Use of Liver Biomechanics in Forensic Pathology. Int. J. Leg. Med..

[104] Van Den Bulcke A.I., Bogdanov B., De Rooze N., Schacht E.H., Cornelissen M., Berghmans H. (2000). Structural and Rheological Properties of Methacrylamide Modified Gelatin Hydrogels. Biomacromolecules.

[105] Rowley J.A., Madlambayan G., Mooney D.J. (1999). Alginate Hydrogels as Synthetic Extracellular Matrix Materials. Biomaterials.

[106] Seremeta K.P., Sosnik A. (2023). Natural and Semi-Natural Polymers.

[107] Parmaksiz M., Dogan A., Odabas S., Elçin A.E., Elçin Y.M. (2016). Clinical Applications of Decellularized Extracellular Matrices for Tissue Engineering and Regenerative Medicine. Biomed. Mater..

[108] Wang J., Qin X., Xia S., Liu S., Ren H. (2023). Orthotopic Implantable Liver Decellularized Scaffold for Acute Liver Failure. Eng. Regen..

[109] Tomofuji K., Fukumitsu K., Kondo J., Horie H., Makino K., Wakama S., Ito T., Oshima Y., Ogiso S., Ishii T. (2022). Liver Ductal Organoids Reconstruct Intrahepatic Biliary Trees in Decellularized Liver Grafts. Biomaterials.

[110] Krüger M., Samsom R.A., Oosterhoff L.A., van Wolferen M.E., Kooistra H.S., Geijsen N., Penning L.C., Kock L.M., Sainz-Arnal P., Baptista P.M. (2022). High Level of Polarized Engraftment of Porcine Intrahepatic Cholangiocyte Organoids in Decellularized Liver Scaffolds. J. Cell Mol. Med..

[111] Willemse J., van Tienderen G., van Hengel E., Schurink I., van der Ven D., Kan Y., de Ruiter P., Rosmark O., Westergren-Thorsson G.G., Schneeberger K. (2022). Hydrogels Derived from Decellularized Liver Tissue Support the Growth and Differentiation of Cholangiocyte Organoids. Biomaterials.

[112] Lewis P.L., Su J., Yan M., Meng F., Glaser S.S., Alpini G.D., Green R.M., Sosa-Pineda B., Shah R.N. (2018). Complex Bile Duct Network Formation within Liver Decellularized Extracellular Matrix Hydrogels. Sci. Rep..

[113] Daneshgar A., Klein O., Nebrich G., Weinhart M., Tang P., Arnold A., Ullah I., Pohl J., Moosburner S., Raschzok N. (2020). The Human Liver Matrisome—Proteomic Analysis of Native and Fibrotic Human Liver Extracellular Matrices for Organ Engineering Approaches. Biomaterials.

[114] van Tienderen G.S., Koerkamp B.G., Ijzermans J.N.M., van der Laan L.J.W., Verstegen M.M.A. (2019). Recreating Tumour Complexity in a Dish: Organoid Models to Study Liver Cancer Cells and Their Extracellular Environment. Cancers.

[115] Carpino G., Overi D., Melandro F., Grimaldi A., Cardinale V., Matteo S.D., Mennini G., Rossi M., Alvaro D., Barnaba V. (2019). Matrisome Analysis of Intrahepatic Cholangiocarcinoma Unveils a Peculiar Cancer-Associated Extracellular Matrix Structure. Clin. Proteom..

[116] Lee J.I., Campbell J.S. (2014). Role of Desmoplasia in Cholangiocarcinoma and Hepatocellular Carcinoma. J. Hepatol..

[117] van Tienderen G.S., Rosmark O., Lieshout R., Willemse J., de Weijer F., Elowsson Rendin L., Westergren-Thorsson G., Doukas M., Groot Koerkamp B., van Royen M.E. (2023). Extracellular Matrix Drives Tumor Organoids toward Desmoplastic Matrix Deposition and Mesenchymal Transition. Acta Biomater..

[118] Milton L.A., Davern J.W., Hipwood L., Chaves J.C.S., McGovern J., Broszczak D., Hutmacher D.W., Meinert C., Toh Y.C. (2024). Liver Click DECM Hydrogels for Engineering Hepatic Microenvironments. Acta Biomater..

[119] Xue T., Zhang J., Li F., Chen G., Yi K., Chen X., Zhang Y., Xu Y., Wang H., Ju E. (2026). Tunable Biomechanical Niches Regulate Hepatic Differentiation of Mesenchymal Stem Cells for Acute Liver Failure Therapy. Biomaterials.

[120] Carpentier N., Ye S., Delemarre M.D., Van der Meeren L., Skirtach A.G., van der Laan L.J.W., Schneeberger K., Spee B., Dubruel P., Van Vlierberghe S. (2024). Gelatin-Based Hybrid Hydrogels as Matrices for Organoid Culture. Biomacromolecules.

[121] Lin C.C., Ki C.S., Shih H. (2015). Thiol-Norbornene Photoclick Hydrogels for Tissue Engineering Applications. J. Appl. Polym. Sci..

[122] Carpentier N., Van der Meeren L., Skirtach A.G., Devisscher L., Van Vlierberghe H., Dubruel P., Van Vlierberghe S. (2023). Gelatin-Based Hybrid Hydrogel Scaffolds: Toward Physicochemical Liver Mimicry. Biomacromolecules.

[123] Bouwmeester M.C., Bernal P.N., Oosterhoff L.A., van Wolferen M.E., Lehmann V., Vermaas M., Buchholz M.B., Peiffer Q.C., Malda J., van der Laan L.J.W. (2021). Bioprinting of Human Liver-Derived Epithelial Organoids for Toxicity Studies. Macromol. Biosci..

[124] Bernal P.N., Bouwmeester M., Madrid-Wolff J., Falandt M., Florczak S., Rodriguez N.G., Li Y., Größbacher G., Samsom R.A., van Wolferen M. (2022). Volumetric Bioprinting of Organoids and Optically Tuned Hydrogels to Build Liver-Like Metabolic Biofactories. Adv. Mater..

[125] Chen Y.X., Cain B., Soman P. (2017). Gelatin Methacrylate-Alginate Hydrogel with Tunable Viscoelastic Properties. AIMS Mater. Sci..

[126] Lipari S., Marfoglia A., Sorrentino G., Cazalbou S., Pilloux L., Sacco P., Donati I. (2025). Thermally Cured Gelatin-Methacryloyl Hydrogels Form Mechanically Modulating Platforms for Cell Studies. Biomacromolecules.

[127] Saito T., Nishiyama Y., Putaux J.L., Vignon M., Isogai A. (2006). Homogeneous Suspensions of Individualized Microfibrils from TEMPO-Catalyzed Oxidation of Native Cellulose. Biomacromolecules.

[128] Syverud K., Pettersen S.R., Draget K., Chinga-Carrasco G. (2015). Controlling the Elastic Modulus of Cellulose Nanofibril Hydrogels—Scaffolds with Potential in Tissue Engineering. Cellulose.

[129] Pääkko M., Ankerfors M., Kosonen H., Nykänen A., Ahola S., Österberg M., Ruokolainen J., Laine J., Larsson P.T., Ikkala O. (2007). Enzymatic Hydrolysis Combined with Mechanical Shearing and High-Pressure Homogenization for Nanoscale Cellulose Fibrils and Strong Gels. Biomacromolecules.

[130] Monfared M., Mawad D., Rnjak-Kovacina J., Stenzel M.H. (2021). 3D Bioprinting of Dual-Crosslinked Nanocellulose Hydrogels for Tissue Engineering Applications. J. Mater. Chem. B.

[131] Krüger M., Oosterhoff L.A., van Wolferen M.E., Schiele S.A., Walther A., Geijsen N., De Laporte L., van der Laan L.J.W., Kock L.M., Spee B. (2020). Cellulose Nanofibril Hydrogel Promotes Hepatic Differentiation of Human Liver Organoids. Adv. Heal. Healthc. Mater..

[132] Yang J., Shao C., Meng L. (2019). Strain Rate-Dependent Viscoelasticity and Fracture Mechanics of Cellulose Nanofibril Composite Hydrogels. Langmuir.

[133] Curvello R., Kerr G., Micati D.J., Chan W.H., Raghuwanshi V.S., Rosenbluh J., Abud H.E., Garnier G. (2021). Engineered Plant-Based Nanocellulose Hydrogel for Small Intestinal Organoid Growth. Adv. Sci..

[134] Amorim S., Reis C.A., Reis R.L., Pires R.A. (2021). Extracellular Matrix Mimics Using Hyaluronan-Based Biomaterials. Trends Biotechnol..

[135] Kim J., Seki E. (2023). Hyaluronan in Liver Fibrosis: Basic Mechanisms, Clinical Implications, and Therapeutic Targets. Hepatol. Commun..

[136] Yang Y.M., Noureddin M., Liu C., Ohashi K., Kim S.Y., Ramnath D., Powell E.E., Sweet M.J., Roh Y.S., Hsin I.F. (2019). Hyaluronan Synthase 2-Mediated Hyaluronan Production Mediates Notch1 Activation and Liver Fibrosis. Sci. Transl. Med..

[137] Petit N., Chang Y.J., Lobianco F.A., Hodgkinson T., Browne S. (2025). Hyaluronic Acid as a Versatile Building Block for the Development of Biofunctional Hydrogels: In Vitro Models and Preclinical Innovations. Mater. Today Bio.

[138] Luo Z., Wang Y., Li J., Wang J., Yu Y., Zhao Y. (2023). Tailoring Hyaluronic Acid Hydrogels for Biomedical Applications. Adv. Funct. Mater..

[139] Collins M.N., Birkinshaw C. (2013). Hyaluronic Acid Based Scaffolds for Tissue Engineering—A Review. Carbohydr. Polym..

[140] Kim Y., Kumar S. (2014). CD44-Mediated Adhesion to Hyaluronic Acid Contributes to Mechanosensing and Invasive Motility. Mol. Cancer Res..

[141] Ananthanarayanan B., Kim Y., Kumar S. (2011). Elucidating the Mechanobiology of Malignant Brain Tumors Using a Brain Matrix-Mimetic Hyaluronic Acid Hydrogel Platform. Biomaterials.

[142] Chopra A., Murray M.E., Byfield F.J., Mendez M.G., Halleluyan R., Restle D.J., Raz-Ben Aroush D., Galie P.A., Pogoda K., Bucki R. (2014). Augmentation of Integrin-Mediated Mechanotransduction by Hyaluronic Acid. Biomaterials.

[143] He Y., Wu G.D., Sadahiro T., Noh S.I., Wang H., Talavera D., Wang H., Vierling J.M., Klein A.S. (2008). Interaction of CD44 and Hyaluronic Acid Enhances Biliary Epithelial Proliferation in Cholestatic Livers. Am. J. Physiol. Gastrointest. Liver Physiol..

[144] Kon J., Ooe H., Oshima H., Kikkawa Y., Mitaka T. (2006). Expression of CD44 in Rat Hepatic Progenitor Cells. J. Hepatol..

[145] Rizwan M., Ling C., Guo C., Liu T., Jiang J.X., Bear C.E., Ogawa S., Shoichet M.S. (2022). Viscoelastic Notch Signaling Hydrogel Induces Liver Bile Duct Organoid Growth and Morphogenesis. Adv. Heal. Healthc. Mater..

[146] Sanz-Horta R., Matesanz A., Gallardo A., Reinecke H., Jorcano J.L., Acedo P., Velasco D., Elvira C. (2023). Technological Advances in Fibrin for Tissue Engineering. J. Tissue Eng..

[147] Broguiere N., Isenmann L., Hirt C., Ringel T., Placzek S., Cavalli E., Ringnalda F., Villiger L., Züllig R., Lehmann R. (2018). Growth of Epithelial Organoids in a Defined Hydrogel. Adv. Mater..

[148] Lu P., Ruan D., Huang M., Tian M., Zhu K., Gan Z., Xiao Z. (2024). Harnessing the Potential of Hydrogels for Advanced Therapeutic Applications: Current Achievements and Future Directions. Signal Transduct. Target. Ther..

[149] Yuan H., Xu J., Van Dam E.P., Giubertoni G., Rezus Y.L.A., Hammink R., Bakker H.J., Zhan Y., Rowan A.E., Xing C. (2017). Strategies to Increase the Thermal Stability of Truly Biomimetic Hydrogels: Combining Hydrophobicity and Directed Hydrogen Bonding. Macromolecules.

[150] Kouwer P.H.J., de Almeida P., ven den Boomen O., Eksteen-Akeroyd Z.H., Hammink R., Jaspers M., Kragt S., Mabesoone M.F.J., Nolte R.J.M., Rowan A.E. (2018). Controlling the Gelation Temperature of Biomimetic Polyisocyanides. Chin. Chem. Lett..

[151] Vandaele J., Louis B., Liu K., Camacho R., Kouwer P.H.J., Rocha S. (2020). Structural Characterization of Fibrous Synthetic Hydrogels Using Fluorescence Microscopy. Soft Matter.

[152] Kouwer P.H.J., Koepf M., Le Sage V.A.A., Jaspers M., Van Buul A.M., Eksteen-Akeroyd Z.H., Woltinge T., Schwartz E., Kitto H.J., Hoogenboom R. (2013). Responsive Biomimetic Networks from Polyisocyanopeptide Hydrogels. Nature.

[153] Das R.K., Gocheva V., Hammink R., Zouani O.F., Rowan A.E. (2016). Stress-Stiffening-Mediated Stem-Cell Commitment Switch in Soft Responsive Hydrogels. Nat. Mater..

[154] Liu K., Mihaila S.M., Rowan A., Oosterwijk E., Kouwer P.H.J. (2019). Synthetic Extracellular Matrices with Nonlinear Elasticity Regulate Cellular Organization. Biomacromolecules.

[155] Ye S., Boeter J.W.B., Mihajlovic M., van Steenbeek F.G., van Wolferen M.E., Oosterhoff L.A., Marsee A., Caiazzo M., van der Laan L.J.W., Penning L.C. (2020). A Chemically Defined Hydrogel for Human Liver Organoid Culture. Adv. Funct. Mater..

[156] Wang Z., Ye S., van der Laan L.J.W., Schneeberger K., Masereeuw R., Spee B. (2024). Chemically Defined Organoid Culture System for Cholangiocyte Differentiation. Adv. Heal. Healthc. Mater..

[157] Lutolf M.P., Lauer-Fields J.L., Schmoekel H.G., Metters A.T., Weber F.E., Fields G.B., Hubbell J.A. (2003). Synthetic Matrix Metalloproteinase-Sensitive Hydrogels for the Conduction of Tissue Regeneration: Engineering Cell-Invasion Characteristics. Proc. Natl. Acad. Sci. USA.

[158] Rezakhani S., Gjorevski N., Lutolf M.P. (2020). Low-Defect Thiol-Michael Addition Hydrogels as Matrigel Substitutes for Epithelial Organoid Derivation. Adv. Funct. Mater..

[159] Kloxin A.M., Kasko A.M., Salinas C.N., Anseth K.S. (2009). Photodegradable Hydrogels for Dynamic Tuning of Physical and Chemical Properties. Science.

[160] Lin C.C., Anseth K.S. (2009). PEG Hydrogels for the Controlled Release of Biomolecules in Regenerative Medicine. Pharm. Res..

[161] McKinnon D.D., Domaille D.W., Cha J.N., Anseth K.S. (2014). Biophysically Defined and Cytocompatible Covalently Adaptable Networks as Viscoelastic 3d Cell Culture Systems. Adv. Mater..

[162] Cruz-Acuña R., Quirós M., Farkas A.E., Dedhia P.H., Huang S., Siuda D., García-Hernández V., Miller A.J., Spence J.R., Nusrat A. (2017). Synthetic Hydrogels for Human Intestinal Organoid Generation and Colonic Wound Repair. Nat. Cell Biol..

[163] Cruz-Acuña R., Quirós M., Huang S., Siuda D., Spence J.R., Nusrat A., García A.J. (2018). PEG-4MAL Hydrogels for Human Organoid Generation, Culture, and in Vivo Delivery. Nat. Protoc..

[164] Gjorevski N., Sachs N., Manfrin A., Giger S., Bragina M.E., Ordóñez-Morán P., Clevers H., Lutolf M.P. (2016). Designer Matrices for Intestinal Stem Cell and Organoid Culture. Nature.

[165] Sorrentino G., Rezakhani S., Yildiz E., Nuciforo S., Heim M.H., Lutolf M.P., Schoonjans K. (2020). Mechano-Modulatory Synthetic Niches for Liver Organoid Derivation. Nat. Commun..

[166] Wang L., Riediger L., Rao Q., Xu X., Nie Y., Zhou Y., Zhang J., Tang P., Wang W., Tacke F. (2025). Tunable Synthetic Hydrogel Modulates Hepatic Lineage Specification of Human Liver Organoid. Adv. Funct. Mater..

[167] Xu R., Ooi H.S., Bian L., Ouyang L., Sun W. (2025). Dynamic Hydrogels for Biofabrication: A Review. Biomaterials.

[168] Zhang Y., Li L., Dong L., Cheng Y., Huang X., Xue B., Jiang C., Cao Y., Yang J. (2024). Hydrogel-Based Strategies for Liver Tissue Engineering. Chem. Bio Eng..

